# Screening Characteristics of TIMI Score in Predicting Acute Coronary Syndrome Outcome; a Diagnostic Accuracy Study

**Published:** 2017-01-09

**Authors:** Mostafa Alavi-Moghaddam, Saeed Safari, Hamideh Alavi-Moghaddam

**Affiliations:** 1 Emergency Department, Imam Hossein Hospital, Shahid Beheshti University of Medical Sciences, Tehran, Iran.; 2 Emergency Department, Shohadaye Tajrish Hospital, Shahid Beheshti University of Medical Sciences, Tehran, Iran.

**Keywords:** Coronary artery disease, prognosis, myocardial infarction, decision support techniques, angina, unstable

## Abstract

**Introduction:**

In cases with potential diagnosis of ischemic chest pain, screening high risk patients for adverse outcomes would be very helpful. The present study was designed aiming to determine the diagnostic accuracy of thrombolysis in myocardial infarction (TIMI) score in Patients with potential diagnosis of ischemic chest pain.

**Method:**

This diagnostic accuracy study was designed to evaluate the screening performance characteristics of TIMI score in predicting 30-day outcomes of mortality, myocardial infarction (MI), and need for revascularization in patients presenting to ED with complaint of typical chest pain and diagnosis of unstable angina or Non-ST elevation MI.

**Results:**

901 patients with the mean age of 58.17 ± 15.00 years (19-90) were studied (52.9% male). Mean TIMI score of the studied patients was 0.97 ± 0.93 (0-5) and the highest frequency of the score belonged to 0 to 2 with 37.2%, 35.3%, and 21.4%, respectively. In total, 170 (18.8%) patients experienced the outcomes evaluated in this study. Total sensitivity, specificity, positive and negative predictive value, and positive and negative likelihood ratio of TIMI score were 20 (95% CI: 17 – 24), 99 (95% CI: 97 – 100), 98 (95% CI: 93 – 100), 42 (95% CI: 39 – 46), 58 (95% CI: 14 – 229), and 1.3 (95% CI: 1.2 – 1.4), respectively. Area under the ROC curve of this system for prediction of 30-day mortality, MI, and need for revascularization were 0.51 (95% CI: 0.47 – 0.55), 0.58 (95% CI: 0.54 – 0.62) and 0.56 (95% CI: 0.52 – 0.60), respectively.

**Conclusion:**

Based on the findings of the present study, it seems that TIMI score has a high specificity in predicting 30-day adverse outcomes of mortality, MI, and need for revascularization following acute coronary syndrome. However, since its sensitivity, negative predictive value, and negative likelihood ratio are low, it cannot be used as a proper screening tool for ruling out low risk patients in ED.

## Introduction

Coronary artery disease (CAD) is a major health problem worldwide (1). Although during the past decades, with development of preventive measures and improvement of diagnostic and therapeutic approaches, CAD related deaths have decreased by more than 40%, CAD still remains the largest killer of the US population (2, 3). About half of all deaths in developed countries and 25% of those in developing ones occur due to CAD (4). It is predicted that until 2020, the number of CAD related deaths will exceed that of infectious diseases (5). It seems that rapid diagnosis, and choosing the best treatment in the initial stages are of great importance in improving the outcome of CAD patients. 

In cases that electrocardiogram (ECG), as the most available and rapid diagnostic tool, is not helpful in decision making (cases of unstable angina and Non-ST elevation MI), screening high risk patients for adverse outcomes would be very helpful. Various clinical decision rules and scoring systems have been developed for this purpose (6, 7).

An ideal model in this regard, should have high sensitivity and be able to predict the outcome accurately, using readily available clinical information. One of these systems is Thrombolysis In Myocardial Infarction (TIMI) scoring model that helps divide the patients into 2 groups of low risk (score 0-1) and high risk (score > 1) regarding adverse 30-day outcome. 

A study on 279 patients visiting ED showed that TIMI score is a valid tool for assessing 30-day mortality risk (8). Additionally, in a study on 3609 ST-elevation MI patients, it was concluded that TIMI score has both short and long term value in predicting patient mortality in those that underwent primary percutaneous coronary intervention (9).

A study by Lee et al. in 2011 in Pennsylvania also revealed the good ability of this system in predicting the incidence of 30-day cardiovascular adverse events following typical chest pain (10). Currently, in many developing countries, supplementary diagnostic measures, such as exercise testing or echocardiography, is not available and cannot be done in EDs. In this situation, using clinical scoring systems may be helpful in patient disposition. Therefore, the present study was designed aiming to determine the diagnostic accuracy of TIMI score in predicting 30-day outcome of patients presenting to ED with diagnosis of unstable angina or Non-ST elevation MI.

## Methods

***Study design***

This study was designed to evaluate the diagnostic accuracy of TIMI clinical scoring system in prediction of 30-day outcome of patients presenting to ED of Imam Hossein Hospital, Tehran, Iran, with potential diagnosis of ischemic chest pain, during a 6-month period (from October 2015 to March 2016). The study was approved by the ethics committee of Shahid Beheshti University of Medical Sciences and the researchers adhered to the principles of Helsinki Declaration and patient data confidentiality throughout the study. A written informed consent was obtained from all the patients for participating in the study.

***Participants***

All the patients with chest pain who were diagnosed with unstable angina or Non-ST elevation MI were entered to the study without any age or sex limitation, using census sampling. Patients with unstable hemodynamics, evidence of ST segment elevation in the initial ECG and those who did not give consent for participating were excluded. Patients with diagnosis of ST elevation MI were immediately scheduled for receiving thrombolytic therapy or percutaneous coronary intervention (PCI).

***Data*** ***gathering***

After learning the study aims and method, the in charge emergency medicine residents were held responsible for data gathering and filling the checklist for the patients on admission. All the patients were relocated to cardiac monitoring unit, and initial life support measures (pain relief with serum nitroglycerin and intravenous morphine, O_2_ therapy, blood pressure monitoring and…) were initiated on admission to ED. In addition, ECG and cardiac enzymes were ordered. All patients underwent 12 lead ECG and in cases suspected to inferior or posterior wall MI, posterior/inferior leads were added. If the pain was not relieved within 20 - 30 minutes after admission, despite initiating therapy with nitroglycerin and intravenous morphine, ECG was repeated with 20 -30 minute intervals up to 3 times. ECG interpretations were done by emergency medicine residents and approved by the in charge attend of the shift and on call internists. Based on the treatment protocol of the studied health center, evaluation of cardiac enzymes including creatine kinase (CK), CKmb, and troponin was also ordered for all the patients on admission and 6 hours later. If the final diagnosis was unstable angina or non-ST elevation MI, all the baseline characteristics of the patient including age and sex, pain characteristics, status of the known risk factors of arteriosclerosis (hypertension, diabetes, smoking, overweight, hyperlipidemia and …), history of cardiovascular diseases (stroke, MI, and…), drugs history, ECG findings, laboratory findings especially level of cardiac enzymes as well as variables needed for calculating TIMI score were gathered by the in charge resident, using a checklist prepared for this purpose. Required data regarding the final outcome of the patients were gathered from their clinical files or, if not available, by phone call with the patient, their relatives, or their cardiologist by a senior emergency medicine resident. Considering the definition of TIMI score the evaluated outcomes including MI, need for revascularization and all-cause mortality during the 30 days after admission to ED (11).

***Statistical analysis***

All analyses were done using SPSS version 20 and STATA 11. Mean, standard deviation, median, range, frequency, and percentage were used to describe data. To calculate the diagnostic accuracy of TIMI system, sensitivity, specificity, positive and negative predictive value, and positive and negative likelihood ratio, and area under the receiver operating characteristic (ROC) curve were calculated.

## Results

***Baseline characteristics of the patients***

901 patients with the mean age of 58.17 ± 15.00 years (19-90) were studied (52.9% male). Table 1 shows the baseline characteristic of the patients. The most common accompanying diseases included hypertension (51.4%) and diabetes (19.8%). ST segment changes (39.2%) and T wave inversion (31.6%) were the most common early ECG findings, respectively.

Table 2 depicts the frequency of factors used in TIMI clinical scoring system among the studied patients. The most common factors in this regard were more than 0.5 mm changes in ST segment (39.2%) and age ≥ 65 years (35.6%). Mean TIMI score of the studied patients was 0.97 ± 0.93 (0-5) and the highest frequency of the score belonged to 0 to 2 with 37.2%, 35.3%, and 21.4%, respectively. Table 3 reveals the final outcome of the participants. 488 (54.2%) of the patients underwent angiography of coronary arteries, based on which 22 (2.4%) had severe obstruction, 371 (41.2%) had moderate obstruction, 72 (8.0%) had mild and 23 (2.6%) had non-significant obstruction.

***Characteristics of TIMI screening system***

In total, 170 (18.8%) patients experienced the outcomes evaluated in this study. Total sensitivity, specificity, positive and negative predictive value, and positive and negative likelihood ratio of TIMI score were 20 (95% CI: 17 – 24), 99 (95% CI: 97 – 100), 98 (95% CI: 93 – 100), 42 (95% CI: 39 – 46), 58 (95% CI: 14 – 229), and 1.3 (95% CI: 1.2 – 1.4), respectively. Table 4 shows the screening characteristics of TIMI score for predicting 30-day mortality, need for revascularization, and MI. The findings reveal the high specificity and low sensitivity of this system in these regards. Area under the ROC curve of this system for prediction of 30-day mortality, MI, and need for revascularization were 0.51 (95% CI: 0.47 – 0.55), 0.58 (95% CI: 0.54 – 0.62) and 0.56 (95% CI: 0.52 – 0.60), respectively.

**Table 1:** Baseline characteristics of the studied patients

**Variable **	**Number (%)**
**Sex **	
Female	424 (47.1)
Male	477 (42.9)
**Risk factors**	
Hypertension	(51.4) 463
Diabetes	178 (19.8)
Smoking	88 (9.8)
Positive family history	29 (3.2)
Hyperlipidemia	88 (9.8)
**Number of accompanying risk factors**	
0	289 (32.1)
1	414 (45.9)
2	164 (18.2)
3	32 (3.6)
4	2 (0.2)
**History of cardiac failure**	26 (2.9)
**PTCA history**	58 (6.4)
**ECG findings**	
ST deviation	353 (39.2)
T inversion	285 (31.6)
Left bundle branch block	28 (3.1)
Right bundle branch block	3 (0.3)
Atrial fibrillation	17 (1.2)
Sinus tachycardia	8 (0.9)
Ventricular tachycardia	1 (0.1)
Multifocal atrial tachycardia	1 (0.1)
Paroxysmal supraventricular tachycardia	6 (0.7)

PTCA: Percutaneous transluminal coronary angioplasty; ECG: electrocardiography.

**Table 2:** Frequency of TIMI risk factors among studied patients (n = 901

**Item **	**Number (%)**
**Age ≥ 65**	321 (35.6)
**≥ 3 CAD risk factors**	34 (3.8)
**Known CAD (stenosis ≥ 50%)**	12 (1.3)
**ASA use in past 7 days**	59 (6.5)
**Sever angina (≥ 2 episodes / 24 hours)**	3 (0.3)
**ST segment changes ≥ 0.5 mm**	353 (39.2)
**Positive cardiac marker**	95 (10.5)

CAD: Coronary artery disease; ASA: Acetylsalicylic acid.

**Table 3:** 30-day outcomes of studied patients

**Outcome**	**Number (%)**
**Myocardial infarction**	88 (9.8)
**Urgent revascularization**	75 (8.3)
Coronary angiography	488 (54.2)
Percutaneous coronary intervention (PCI)	332 (36.8)
Coronary artery bypass graft (CABG)	34 (3.8)
**Admission to coronary care unit (CCU)**	712 (79.0)
**Death**	7 (0.8)

**Table 4:** Screening performance characteristics of TIMI score in 30-day outcome prediction of patients with unstable angina/non-ST elevation myocardial infarction (MI

**Outcomes**	**Mortality**	**MI**	**Revascularization**
**Sensitivity**	1.2 (0.5-2.6)	15 (12-18)	13 (10-16)
**Specificity**	100 (98-100)	100 (98-100)	99 (97-99)
**Positive predictive value**	100 (56-100)	100 (94-100)	97 (89-99)
**Negative predictive value**	38 (35-42)	41 (37-44)	40 (36-43)
**Positive likelihood ratio**	∞	∞	37 (9-143)
**Negative likelihood ratio**	1.5 (1.4-1.6)	1.4 (1.3-1.5)	1.5 (1.4-1.6)

Data were presented with 95% confidence interval.

## Discussion

Findings of the present study, reveal the high specificity (99%) of TIMI score in prediction of 30-day clinical outcomes of mortality, MI, and need for revascularization. However, it has a low sensitivity (1.2 – 15%) and its screening power is low. 

A study by Marcoon et al. in 2013 in America showed that the probability of adverse 30-day outcome in patients with typical chest pain and 0 TIMI score is lower than 1% (12). In addition, a cohort study by Pollack et al. revealed that the risk of short term adverse outcome is 2.1% in patients with 0 TIMI score and 100% in those with a score higher than 7 (13). In a meta-analysis, Hess et al. expressed that there is 1.8% risk of 30-day cardiac events in those with 0 TIMI score, while the risk is 4% for those with a score of 1. They found a strong linear correlation between TIMI score and incidence of cardiac events (14). 

In a cohort study in 30, Lee et al. concluded that patients with high risk of adverse outcome may be identified in ED using this system (10). This result is completely in line with the findings of the present study since the high specificity of this model makes it a useful tool to rule in at risk patients. On the other hand, low sensitivity, negative predictive value and negative likelihood ratio has limited its use for screening and ruling out high risk patients in ED. However, this tool can be at least used for preventing discharge of high risk patients from ED.

In the meta-analysis by Hess et al. that included 8 studies, at >0 cut off, sensitivity was calculated to be 97.2%, specificity was 25.0%, and negative likelihood ratio was 0.11. They concluded that patients should not be discharged solely because of low TIMI score (14). The findings of the preset study also showed the low sensitivity (20%) and high specificity (99%) of TIMI score.

 Jain et al. compared the prognostic value of TIMI score with HEART score in predicting 30-day and 5-year outcomes of mortality, MI, and revascularization, and concluded that HEART score is superior (19). This finding was in line with other studies that expressed HEART score has higher discriminatory power compared to TIMI score (20, 21). It has also been found that Global Registry of Acute Coronary Events (GRACE) risk score has significantly higher discriminatory accuracy in comparison with TIMI score (22). In comparison of HEART, TIMI, and GRACE scores, HEART score is found to be the best of the 3 (23). A study compared Emergency Department Assessment of Chest Pain Score (EDACS), with HEART and TIMI scores in prediction of 6-week major adverse cardiovascular events of MI, revascularization and death, in a 6 week follow-up period for patients with chest pain presenting to ED. It was revealed that EDACS score was able to identify more low-risk patients compared to the other 2 scores (24). Modified Goldman risk score in combination with high-sensitivity troponin was also found to identify significantly more low-risk patients for 30-day adverse outcomes compared to TIMI score (25). CHADS_2_ score has also been deemed more practical for outcome prediction of acute MI patients (26). Some studies even expressed that TIMI score does not have a predictive capacity, since its area under the ROC curve is 0.532, which is close to the area under the curve found in this study (27).

Among the limitations of this study is extraction of outcome from patients’ medical profile might leading to miss some important points, however, we tried to minimize this probability by contacting the patient or their in-charge cardiologist.

## Conclusion:

 Based on the findings of the present study, it seems that TIMI score has a high specificity in predicting 30-day adverse outcomes of mortality, MI, and need for revascularization following acute coronary syndrome. However, since its sensitivity, negative predictive value, and negative likelihood ratio are low, it cannot be used as a proper screening tool for ruling out low risk patients in ED.
